# Extensive Iliofemoral Deep Vein Thrombosis Secondary to Uterine Leiomyoma-Induced Iliac Vein Compression: A Case Report

**DOI:** 10.7759/cureus.114364

**Published:** 2026-08-11

**Authors:** Noor Albaraq, Hasan J Hamam

**Affiliations:** 1 Family Medicine, Detroit Medical Center Sinai-Grace Hospital, Detroit, USA

**Keywords:** deep vein thrombosis (dvt), family medicine residency, giant uterine leiomyoma, iliac vein compression, iliofemoral deep vein thrombosis

## Abstract

Deep vein thrombosis (DVT) is an acute vascular condition commonly associated with immobility, hypercoagulability, and endothelial injury. We report the case of a 47-year-old woman presenting with progressive left lower extremity swelling and pain, ultimately diagnosed with extensive iliofemoral DVT secondary to extrinsic compression of the left common iliac vein by a markedly enlarged fibroid uterus. The patient was managed with therapeutic anticoagulation, blood transfusion, and intravenous iron therapy, followed by definitive hysterectomy approximately two months later, with guideline-directed perioperative apixaban interruption and resumption without bridging anticoagulation. This case highlights the importance of cross-sectional imaging in identifying pelvic mass effect as an underrecognized cause of extensive unilateral DVT and the need for definitive treatment of the underlying compressive pathology.

## Introduction

Deep vein thrombosis (DVT) typically results from the classic Virchow triad: venous stasis, hypercoagulability, and endothelial injury. Anatomical causes, such as May-Thurner syndrome (compression of the left common iliac vein by the right common iliac artery), are well recognized. However, extrinsic venous compression from pelvic masses, such as uterine leiomyomas, is a rare but clinically significant cause [1,2].

Uterine leiomyomas are the most common benign tumor in women of reproductive age, with the majority remaining asymptomatic [1]. Despite that, large fibroids can exert a mass effect on adjacent pelvic structures. Venous thromboembolism (VTE) secondary to fibroid-induced venous compression is uncommon, with a recent systematic review identifying only 24 documented cases [1]. The mechanism involves mechanical compression of pelvic veins, most commonly the left iliac vein, leading to venous stasis and subsequent thrombosis [2,3].

Recognizing this association is clinically important, particularly in patients presenting with extensive unilateral lower-extremity DVT in the absence of traditional risk factors. We present a case of extensive iliofemoral DVT secondary to iliac vein compression by a large fibroid uterus, successfully managed with anticoagulation and hysterectomy.

## Case presentation

A 47-year-old woman with a known history of uterine fibroids presented to the emergency department with progressive left lower-extremity swelling and pain. Two days before presentation, she experienced mild left knee tenderness. The following day, the left leg developed a sensation of tightness and heaviness. She used compression socks and rested overnight but awoke with increased pain and noted a noticeable difference in leg size compared with the right. Symptoms progressed to include pins and needles, numbness, and heaviness in the left foot, requiring assistance to ambulate. Swelling was initially limited to the knee but later extended proximally.

She denied fever, chills, chest pain, shortness of breath, palpitations, lightheadedness, dizziness, or syncope. She reported a remote episode of illness around Christmas that had resolved. She denied recent long-distance travel except for a trip to Hawaii four weeks ago, which involved more than 10 hours in flight, and a previous trip to France in September. She reported heavy menstrual periods lasting six to seven days. Past medical history was significant for uterine fibroids. Family history was notable for deep vein thrombosis in her father at approximately the same age, occurring in 2003 and 2005, and for lung cancer in her maternal grandfather. Social history was notable for former tobacco use in college (none in the last 20 years), employment as an attorney with prolonged sitting, and no reported alcohol or illicit substance use.

Physical examination revealed a markedly swollen, erythematous, and tender left lower extremity with a significant difference in circumference compared with the right. Pedal pulses were palpable bilaterally, and neurovascular status was intact. Abdominal examination revealed a firm, non-tender pelvic mass. Vital signs were stable with no evidence of hemodynamic compromise.

Laboratory evaluation on admission revealed significant microcytic hypochromic anemia with hemoglobin of 7.9 g/dL, MCV of 65.5 fL, and MCHC of 29.6%, consistent with chronic iron deficiency secondary to menorrhagia. White blood cell count was mildly elevated at 13.0 × 10^3^/mm^3^ with neutrophilic predominance (87%). Coagulation studies showed no clinically significant coagulopathy (PT: 10.4 seconds, INR: 0.97, aPTT: 23.1 seconds). The comprehensive metabolic panel revealed no clinically significant metabolic abnormalities (Table 1).

**Table 1:** Summary of patient's laboratory values on admission MCV: mean corpuscular volume; MCH: mean corpuscular hemoglobin; MCHC: mean corpuscular hemoglobin concentration; RDW: red cell distribution width; WBC: white blood cell; RBC: red blood cell; BUN: blood urea nitrogen; eGFR: estimated glomerular filtration rate; PT: prothrombin time; INR: international normalized ratio; aPTT: activated partial thromboplastin time; CKD-EPI: Chronic Kidney Disease Epidemiology Collaboration (a mathematical formula used to estimate the kidney filtration known as eGFR)

Test	Patient value	Reference range
Hemoglobin	7.9 g/dL	12.0-16.0 g/dL
Hematocrit	26.70%	36.0-46.0%
MCV	65.5 fL	80-100 fL
MCH	19.4 pg	27-33 pg
MCHC	29.60%	32-36%
RDW	19.70%	11.5-14.5%
WBC	13.0 × 10^3^/mm^3^	4.0-11.0 × 10^3^/mm^3^
Platelets	315 × 10^3^/mm^3^	150-400 × 10^3^/mm^3^
RBC morphology	Moderate anisocytosis	-
Sodium	136 mMol/L	135-145 mMol/L
Potassium	3.7 mMol/L	3.5-5.0 mMol/L
Chloride	102 mMol/L	98-107 mMol/L
CO₂	21 mMol/L	22-29 mMol/L
BUN	18 mg/dL	7-20 mg/dL
Creatinine	0.88 mg/dL	0.6-1.3 mg/dL
eGFR (CKD-EPI)	81 mL/min/1.73 m²	>60 mL/min/1.73 m²
Glucose	135 mg/dL	70-110 mg/dL
Calcium	9.0 mg/dL	8.5-10.5 mg/dL
Magnesium	1.9 mg/dL	1.7-2.2 mg/dL
PT	10.4 seconds	11.0-13.5 seconds
INR	0.97	0.8-1.2
aPTT	23.1 seconds	25.0-35.0 seconds

**Figure 1:**
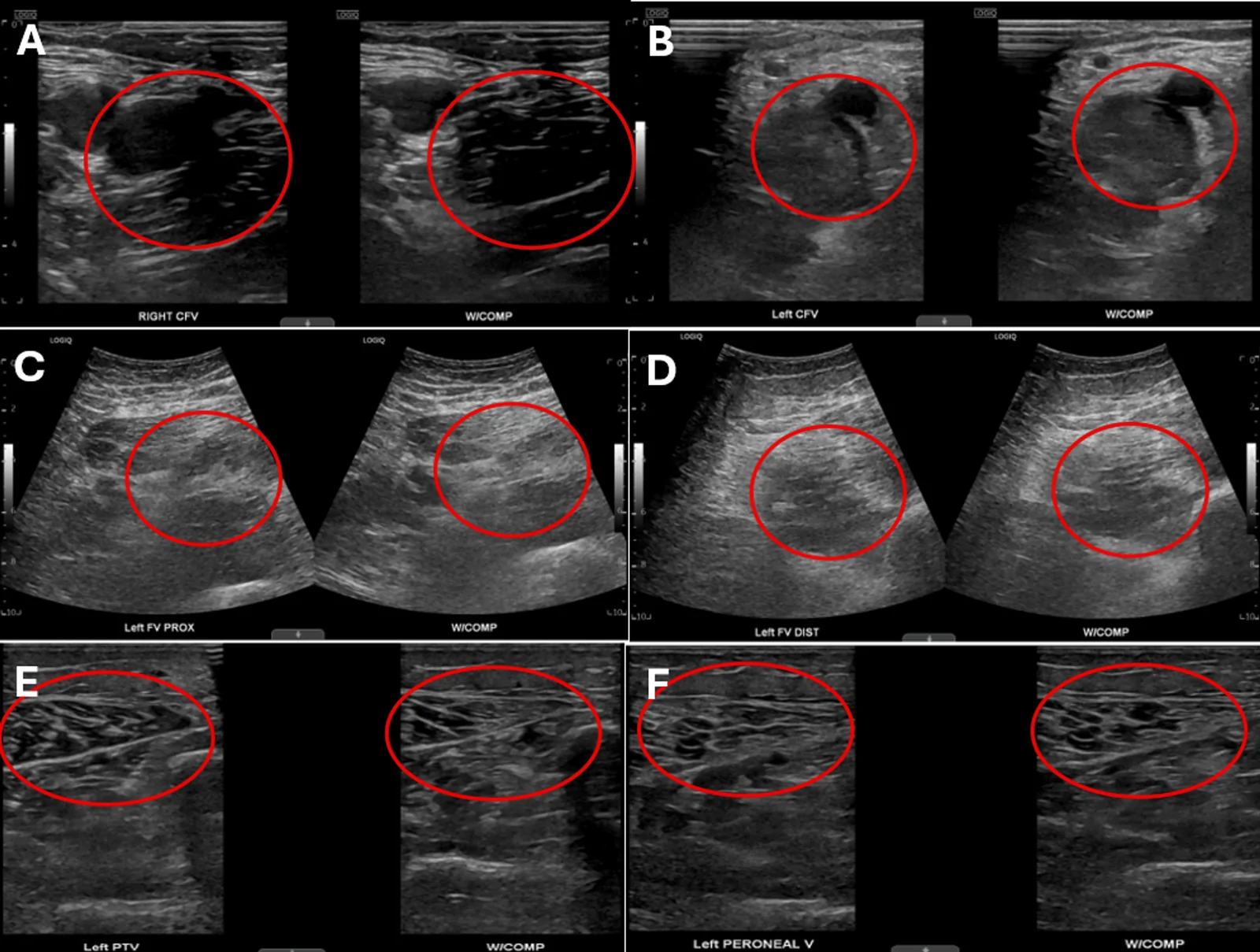
Venous duplex ultrasound compression study of the bilateral lower extremities (A) Right common femoral vein (CFV) without compression, demonstrating a patent lumen (red circle). Right CFV with compression (W/COMP), showing complete venous collapse, confirming the absence of thrombus (red circle). (B) Left CFV without compression, demonstrating a distended vein consistent with acute thrombus (red circle). Left CFV with compression, showing non-compressibility of the vein, confirming acute deep vein thrombosis (red circle). (C) Left proximal femoral vein (FV PROX) without compression, showing intraluminal thrombus with loss of normal lumen (red circle). Left FV PROX with compression, demonstrating persistent non-compressibility (red circle). (D) Left distal femoral vein (FV DIST) without compression consistent with thrombus extension (red circle). Left FV DIST with compression, confirming non-compressibility (red circle). (E) Left posterior tibial vein (PTV) without compression, demonstrating patent veins (red circle). Left PTV with compression, showing complete venous collapse, confirming the absence of calf vein thrombosis (red circle). (F) Left peroneal vein without compression, demonstrating patent paired veins (red circle). Left peroneal vein with compression, showing complete venous collapse, confirming no thrombus extension into the calf veins (red circle). Images for aortoiliac, IVC, and popliteal veins duplex ultrasound were not included due to quality issues. IVC: inferior vena cava

Venous duplex ultrasound of the left lower extremity demonstrated acute DVT involving the left common femoral, femoral, and popliteal veins (Figure 1). The contralateral common femoral vein was patent. Aortoiliac and inferior vena cava (IVC) duplex showed a patent IVC and iliac veins with known left common femoral DVT. Figure 1 presents selected representative images rather than all thrombosed segments, as radiology confirmed the presence of thrombosis in the left common iliac vein (figure not included).

**Figure 2:**
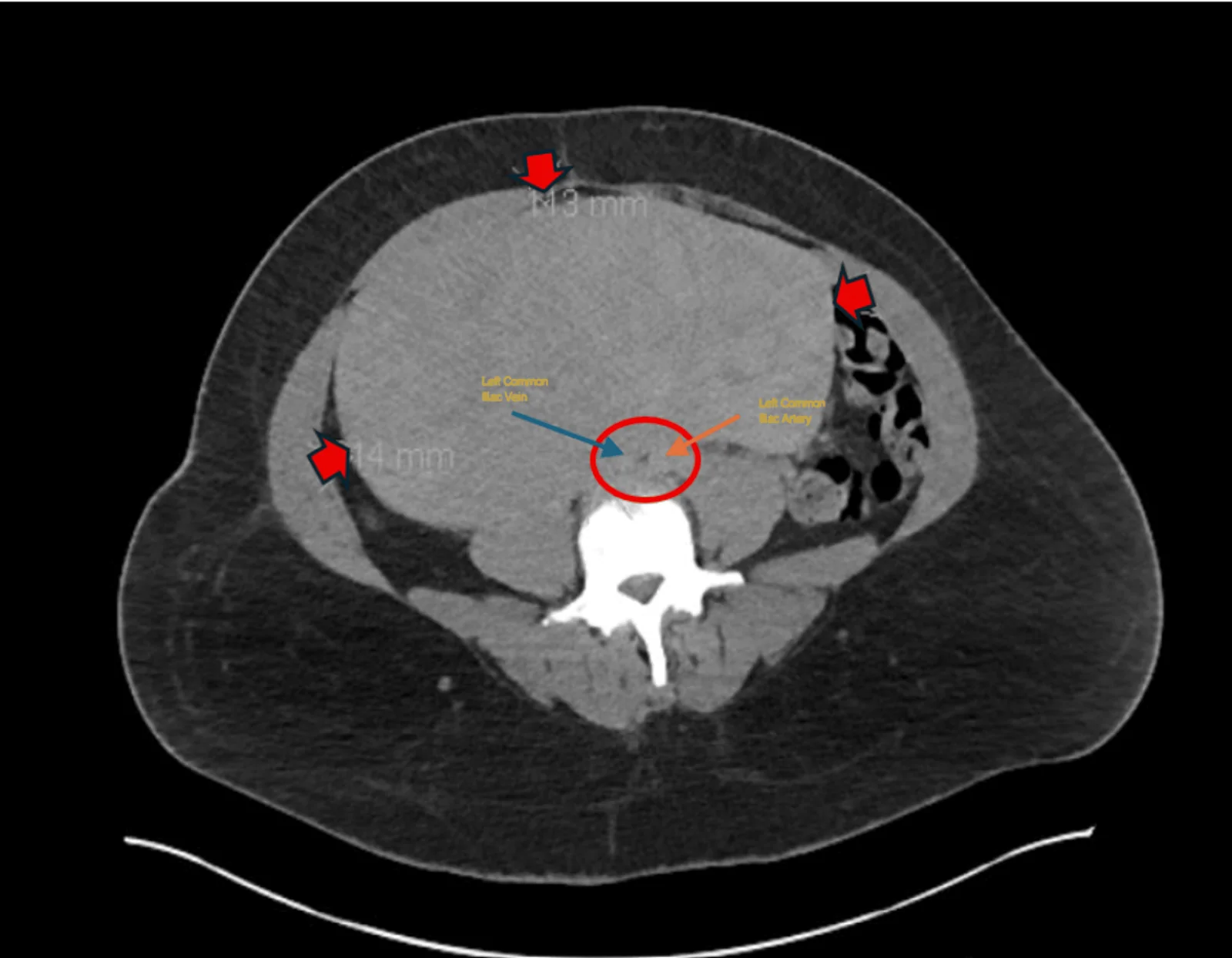
Computed tomography venography of the abdomen and pelvis demonstrating extrinsic compression of the left common iliac vein by the markedly enlarged fibroid uterus with associated distal venous engorgement. Figure shows a markedly enlarged, heterogeneous uterus (red arrows, 21 × 11 × 23 cm) with multiple leiomyomas. The red circle highlights the left common iliac vein (blue arrow) and left common iliac artery (orange arrow). The left common iliac vein is displaced medially by the uterine mass effect and compressed against the vertebral body and the artery. Both iliac veins are crowded together rather than in their normal anatomic positions, reflecting severe extrinsic venous compression. Asymmetric soft tissue edema of the left thigh is noted, consistent with distal venous congestion. A thrombus was seen in the left common iliac vein; however, it is not visualized in this CTV image. CTV: computed tomography venography

Computed tomography venography (CTV) of the abdomen and pelvis revealed significant extrinsic compression of the left common iliac vein by a markedly enlarged fibroid uterus, resulting in distal venous engorgement and edema, consistent with severe venous stenosis from uterine mass effect rather than May-Thurner syndrome (Figure 2). There were no clinical signs or symptoms of pulmonary embolism.

**Figure 3:**
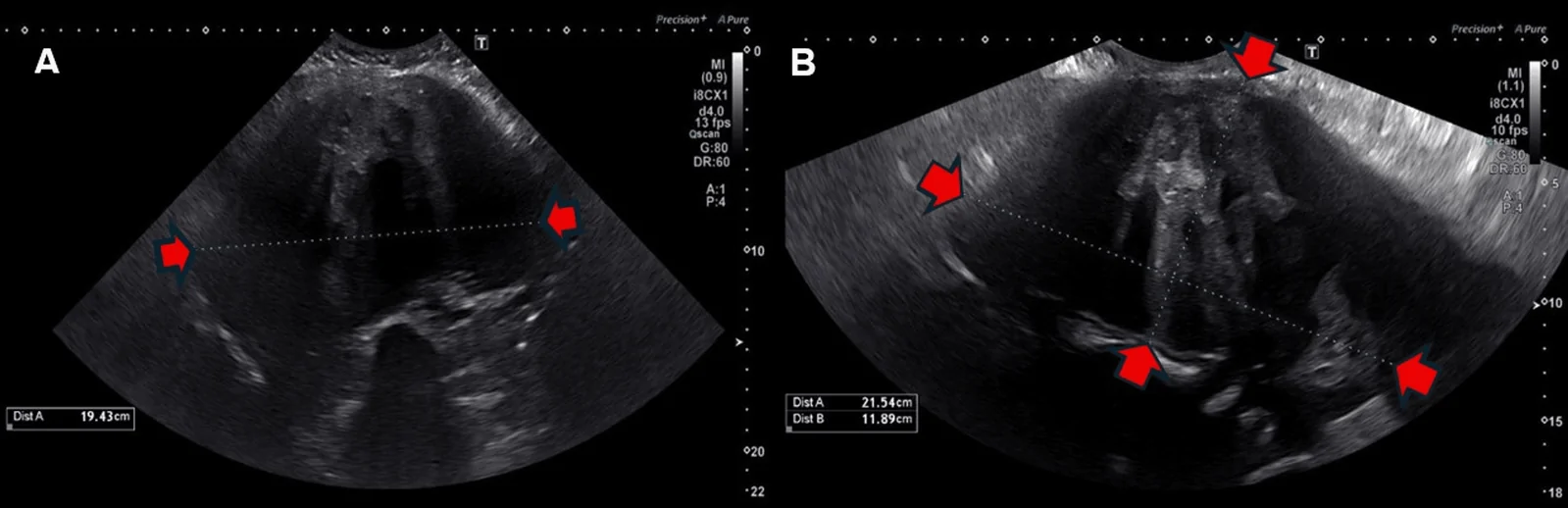
Pelvic ultrasound demonstrating a markedly enlarged anteverted uterus. The figure shows the uterus measuring 21.5 × 11.9 × 19.5 cm (indicated by the red arrows) with multiple leiomyomas. (A) Transverse width of the uterus measuring 19.5 cm. (B) Superior-inferior length of the uterus measuring 21.5 cm and anterior-posterior depth measuring 11.9 cm.

Pelvic ultrasound confirmed an anteverted, enlarged uterus measuring 21.5 × 11.9 × 19.5 cm with multiple large uterine masses, including a heterogeneous mass measuring 9.4 × 6.3 × 8.8 cm, a second myometrial mass measuring 4.2 × 3.9 × 4.2 cm, and a third myometrial mass measuring 6.7 × 5.5 × 6.3 cm. The endometrium was poorly visualized secondary to the multiple masses, and neither ovary was visualized secondary to the enlarged uterus. No pelvic fluid was identified (Figure 3).

The initial differential diagnosis included May-Thurner syndrome, hypercoagulable state, malignancy-associated thrombosis, and immobility-related thrombosis. CTV identified severe extrinsic compression by the fibroid uterus as the primary provoking mechanism, consistent with severe venous stenosis from uterine mass effect rather than May-Thurner syndrome [1,2,4].

The patient was started on a heparin drip, with improvement in pain and preservation of sensorimotor function and distal Doppler signals. Vascular surgery was consulted and recommended no acute surgical intervention.

Given her age, the extensive iliofemoral thrombus, and her family history, a thrombophilia evaluation was pursued. Functional protein C, protein S, and antithrombin assays were deliberately deferred rather than omitted, as both acute thrombosis and heparin render them uninterpretable: heparin falsely lowers antithrombin activity and may falsely normalize protein S and PTT-based protein C activity. Factor V Leiden was tested during admission because PCR genotyping is unaffected by anticoagulation; the result was negative. ANA was obtained as a screen for underlying connective tissue disease, given her age and menorrhagia, and was negative; it is not part of an inherited thrombophilia panel, and antiphospholipid antibody testing (lupus anticoagulant, anticardiolipin, anti-β2-glycoprotein I) was deferred to the outpatient setting given the high false-positive rate during acute thrombosis and anticoagulation. Serum protein electrophoresis (SPEP) was obtained to evaluate the anemia rather than as part of the thrombophilia workup and showed hypoalbuminemia without a monoclonal protein.

Hemoglobin trended downward to a nadir below 7.0 g/dL, prompting transfusion of two units of packed red blood cells with stabilization to 8.3 g/dL. She also had an episode of heavy menstrual bleeding during admission, and iron studies were consistent with iron deficiency anemia. She received intravenous iron before discharge and was started on oral iron therapy. She was referred to hematology for completion of the evaluation, with functional natural anticoagulant and antiphospholipid testing planned once interpretable off anticoagulation.

From a vascular standpoint, the patient was cleared to transition from heparin to apixaban, with a one-week 10 mg twice-daily loading dose followed by 5 mg twice daily for at least three months [5]. Gynecology was consulted; no acute gynecologic intervention was indicated as the patient was not actively bleeding at the time of evaluation. Given her age of more than 45 years with previous abnormal uterine bleeding, outpatient endometrial sampling was recommended to rule out malignancy before definitive management. The patient was counseled extensively on the importance of follow-up and agreed to the plan for outpatient workup and long-term management, including eventual hysterectomy once medically cleared in the setting of current DVT.

The patient was discharged in stable condition on apixaban, with outpatient follow-up arranged with gynecology and benign hematology. Approximately two months after discharge, she underwent a total hysterectomy at an outside institution. Per available records, apixaban was held two days before surgery, consistent with the American College of Chest Physicians (ACCP) recommendation to discontinue apixaban at least 48 hours before high bleeding risk procedures [6,7]. Heparin bridging was not administered, as current guidelines recommend against perioperative bridging anticoagulation during direct oral anticoagulant (DOAC) interruption given the rapid offset and onset of these agents. An inferior vena cava filter was not placed, as the patient had been therapeutically anticoagulated for approximately two months and met no guideline-based indication for filter placement. Apixaban was resumed at therapeutic dosing on postoperative day 2, once adequate hemostasis was confirmed, consistent with ACCP recommendations to delay DOAC resumption 48-72 hours after a high bleeding risk surgery. No perioperative thrombotic or hemorrhagic complications occurred. Pathology confirmed benign leiomyomas with no evidence of malignancy (biopsy was acquired during hysterectomy). At follow-up, the patient reported significant improvement in left lower extremity swelling and pain with return to independent ambulation. Anticoagulation was continued for a minimum of three months per VTE treatment guidelines [5].

It is important to note that no follow-up venous duplex or cross-sectional imaging was available to evaluate for residual thrombosis, recanalization, or persistent venous obstruction following anticoagulation and hysterectomy, limiting the objective assessment of treatment response and long-term vascular outcomes.

## Discussion

This case illustrates a rare but clinically significant cause of extensive DVT: extrinsic compression of the iliac vein by a large fibroid uterus. Although uterine fibroids are extremely common, VTE as a complication is rare. A recent systematic review by Sparić et al. identified only 24 documented cases of VTE associated with uterine fibroids [1]. A 2021 case series by Cai et al. found that among 35 patients with fibroid-associated VTE, the average uterine size was 22.9 weeks gestational age, 60% of thromboses occurred on the left side, and 87% showed venous compression on imaging [2]. These findings align with our patient's presentation.

The mechanism of thrombosis in this setting involves mechanical compression of pelvic veins by the enlarged uterus, creating venous stasis and triggering the thrombotic cascade [1-3]. This mimics the physiology of May-Thurner syndrome but differs in etiology. Rather than arterial compression of the vein, the compression is from an extrinsic pelvic mass [4,7]. The left-sided predominance may reflect the anatomical position of the left common iliac vein, which is relatively fixed between the right common iliac artery anteriorly and the vertebral body posteriorly [2,7].

Our patient had several additional risk factors that may have contributed to thrombosis, including a strong family history of DVT in her father at a similar age, prolonged sitting as an attorney, and a recent long-distance trip to Hawaii. The combination of mechanical venous compression with these prothrombotic factors likely acted synergistically to precipitate extensive thrombosis. Notably, the systematic review by Sparić et al. found that none of the 24 reviewed cases had confirmed inherited thrombophilia, though testing was often incomplete [1]. In our patient, the hypercoagulable workup was limited by active heparin infusion, and complete thrombophilia testing was deferred to the outpatient setting.

Cross-sectional imaging with CT or MRI is essential for patients presenting with extensive unilateral DVT, particularly when traditional risk factors are absent [1,2]. Although venous duplex ultrasound is the primary diagnostic modality for DVT, it has limited ability to visualize pelvic veins above the inguinal ligament [4]. In our patient, CTV was crucial for identifying the compressive etiology and demonstrating severe venous stenosis from uterine mass effect. Distinguishing fibroid-induced compression from true May-Thurner syndrome is clinically important because management strategies differ: May-Thurner syndrome may be treated with endovascular stenting, whereas fibroid-induced compression requires treatment of the underlying pelvic mass [1,7-8].

An important consideration in this case was the need for endometrial sampling before definitive surgery. Given the patient's age of more than 45 years and persistent abnormal uterine bleeding, an endometrial biopsy was recommended to rule out endometrial malignancy before hysterectomy, in accordance with current gynecologic guidelines [7].

Management of fibroid-associated DVT requires a dual approach: anticoagulation to treat the acute thrombosis and definitive treatment of the underlying compressive pathology [1-3,9]. In the systematic review by Sparić et al., nearly half of patients underwent hysterectomy, which was the most successful treatment strategy [1]. Cai et al. reported that 89% of patients required surgical management to relieve venous compression [2]. A recent case report by Qammar et al. described successful management with mechanical aspiration thrombectomy, iliac vein stenting, and hysterectomy [10]. Alternative approaches such as myomectomy may be considered in patients desiring uterine preservation, though data are limited.

Patients with recent VTE undergoing surgery pose unique challenges for perioperative anticoagulation management. Careful perioperative planning that addresses both thrombotic and bleeding risks is essential in this population [6-7]. The ACCP guidelines recommend a standardized approach to perioperative DOAC management, with apixaban held for two to three days preoperatively, based on bleeding risk. Heparin bridging for DOAC-treated patients is generally not recommended [6-7].

This case underscores several important clinical points. First, pelvic masses, such as uterine fibroids, can compress the venous system and lead to DVT. Clinicians should maintain a high index of suspicion for anatomical causes in patients with extensive unilateral thrombosis [1-2]. Second, extrinsic compression may mimic May-Thurner syndrome, and cross-sectional imaging is essential to distinguish between these entities [4,7,9]. Third, definitive management requires treatment of the underlying cause alongside anticoagulation [1-3,10]. Finally, a multidisciplinary approach involving vascular surgery, gynecology, and hematology is critical for optimizing outcomes in these complex patients.

## Conclusions

Extrinsic compression of the iliac vein by large uterine fibroids is a rare but important cause of extensive DVT. Clinicians should maintain a low threshold for cross-sectional imaging in patients presenting with unilateral lower-extremity DVT, particularly when traditional risk factors are absent or pelvic pathology is suspected. Management requires therapeutic anticoagulation and definitive treatment of the underlying pelvic mass, with hysterectomy or another appropriate gynecologic intervention depending on the clinical context. This case highlights the importance of recognizing pelvic masses as potential contributors to venous obstruction and the need for a multidisciplinary approach to optimize patient outcomes.
